# Notch2 Controls Prolactin and Insulin-Like Growth Factor Binding Protein-1 Expression in Decidualizing Human Stromal Cells of Early Pregnancy

**DOI:** 10.1371/journal.pone.0112723

**Published:** 2014-11-14

**Authors:** Gerlinde R. Otti, Leila Saleh, Philipp Velicky, Christian Fiala, Jürgen Pollheimer, Martin Knöfler

**Affiliations:** 1 Department of Obstetrics and Fetal-Maternal Medicine, Reproductive Biology Unit, Medical University of Vienna, Vienna, Austria; 2 Gynmed Clinic, Vienna, Austria; Michigan State University, United States of America

## Abstract

Decidualization, the transformation of the human uterine mucosa into the endometrium of pregnancy, is critical for successful implantation and embryonic development. However, key regulatory factors controlling differentiation of uterine stromal cells into hormone-secreting decidual cells have not been fully elucidated. Hence, we herein investigated the role of the Notch signaling pathway in human decidual stromal cells (HDSC) isolated from early pregnancy samples. Immunofluorescence of first trimester decidual tissues revealed expression of Notch2 receptor and its putative, membrane-anchored interaction partners Jagged1, Delta-like (DLL) 1 and DLL4 in stromal cells whereas other Notch receptors and ligands were absent from these cells. During in vitro differentiation with estrogen/progesterone (E2P4) and/or cyclic adenosine monophosphate (cAMP) HDSC constitutively expressed Notch2 and weakly downregulated Jagged1 mRNA and protein, measured by quantitative PCR (qPCR) and Western blotting, respectively. However, increased levels of DLL1 and DLL4 were observed in the decidualizing cultures. Transfection of a Notch luciferase reporter and qPCR of the Notch target gene hairy and enhancer of split 1 (HES1) revealed an induction of canonical Notch activity during in vitro differentiation. In contrast, treatment of HDSC with a chemical Notch/γ-secretase inhibitor decreased cAMP/E2P4-stimulated Notch luciferase activity, HES1 transcript levels and mRNA expression of the decidual marker genes prolactin (PRL) and insulin-like growth factor binding protein 1 (IGFBP1). Similarly, siRNA-mediated gene silencing or antibody-mediated blocking of Notch2 diminished HES1, PRL and IGFBP1 mRNA levels as well as secreted PRL protein. In summary, the data suggest that canonical, Notch2-dependent signaling plays a role in human decidualization.

## Introduction

Implantation is a highly complex event that requires coordinated interactions of the blastocyst with the receptive endometrium. Rising progesterone concentrations in the second half of the menstrual cycle in conjunction with elevated cyclic adenosine monophosphate (cAMP) levels initiate profound biochemical and morphological changes of estrogen-primed endometrial cells, a process termed decidualization [1], [2]. In humans, decidual differentiation starts prior to conception. However, the process is terminated in the absence of pregnancy, due to the atrophying corpus luteum leading to shedding of the endometrial mucosa at the end of the menstrual cycle. Hence, complete decidualization needs signals emanating from the implanting blastocyst, thereby creating a unique spatio-temporal milieu which protects endometrial cells from insults such as inflammatory signals or oxidative stress [1], [3], [4]. Differentiation affects all cell types of the endometrium, such as growth and coiling of the spiral arteries, alterations of glandular secretion, influx of uterine natural killer (uNK) cells and macrophages as well as decidualization of the stromal compartment [1]. The latter is accompanied by characteristic cellular changes, for example acquisition of an epithelial-like phenotype, expression of specific transcription factors and secretion of proteins associated with differentiation such as prolactin (PRL) and insulin-like growth factor binding protein 1 (IGFBP1) [1], [5]. Furthermore, decidualization likely provides the basis for subsequent placental development, since factors secreted from decidual glands and stromal cells regulate multiple cellular processes at the feto-maternal interface such as trophoblast proliferation, invasion or recruitment of different immune cells [1], [6]. Hence, formation of a functional decidua is indispensable for successful progression of pregnancy, failures in this process have been associated with infertility and miscarriage [1], [7]. Despite these facts, key regulatory genes and developmental signaling pathways controlling decidualization have only partly been unraveled.

Notch signaling is known to regulate diverse cellular functions such as maintenance and differentiation of stem cells, adhesion, invasion, and cell survival [8], [9]. Vertebrates express four different Notch receptors (Notch1 to 4), operating both at the surface of cells to receive signals and in the nucleus to act as transcriptional modulators [10]. Notch receptors are single-pass transmembrane proteins consisting of a functional Notch extracellular domain (NECD) non-covalently linked to a transmembrane and a Notch intracellular domain (NICD). Activation of canonical Notch signaling is initiated by direct interaction of a Notch receptor with one of the five membrane-anchored Delta Serrate LAG2 (DSL) ligands, Delta-like (DLL) 1, 3, 4, Jagged 1 or 2, on two adjacent cells. Formation of the receptor-ligand complex triggers two proteolytic cleavages of Notch by A Distegrin And Metalloprotease (ADAM) and γ-secretase, provoking the release of NICD to the cytoplasm. After translocation to the nucleus, the NICD binds to recombination signal binding protein for immunoglobulin kappa J region (RBPJκ), thereby converting the key regulatory transcription factor of canonical Notch signaling into an activator. After recruitment of additional co-activators, RBPJκ induces canonical Notch targets such as hairy and enhancer of split (HES) and hairy and enhancer of split-related (HEY) genes, which act as transcriptional repressors [10], [11].

Recent investigations provided evidence that Notch family members are differentially expressed in the human cycling endometrium. While Notch4 is strongly expressed in the estrogen-dominated first half of the menstrual cycle, the proliferative phase, Notch1 and Jagged1 are more abundant during the second half of the cycle, the secretory phase [12]. Along those lines, Notch1-4, Jagged1 and DLL4 mRNAs have been detected in endometrial epithelial and stromal cells and DLL4 expression was elevated during the late proliferative and early secretory phase [13], [14], [15]. In pregnant baboon, human chorionic gonadotrophin (hCG)-induced Notch1 was shown to prevent apoptosis of endometrial stromal cells which could suppress menstrual shedding during early decidualization [16]. However, downregulation of Notch1 and induction of the Notch inhibitor Numb could be necessary for complete differentiation of human uterine fibroblasts into decidual cells [17]. Additionally, expression of Notch receptors on uNK cells could play a role in the cross-talk with other ligand-producing cell types of the uterine environment [18]. Moreover, Notch3 and 4 mRNAs were shown to be downregulated in the decidua of preeclamptic pregnancies [19], [20]. Hence, the Notch signaling pathway could play a critical role in physiological and aberrant endometrial and decidual function. However, distribution of all Notch receptors and ligands in uterine tissues of early human pregnancy and their individual roles in decidualization still remain to be elucidated. Therefore, we herein investigated the expression pattern and function of the particular signaling pathway in human decidual stromal cells (HDSC) isolated form early pregnancy tissue. As recently shown, in vitro cultivation of these cells in the presence of estrogen/progesterone (E2P4) and cAMP promotes differentiation and expression of the decidual marker genes PRL and IGFBP1 [21].

## Materials and Methods

### Tissue collection and isolation of primary human decidual stromal cells

Human decidual specimens (n = 23) were obtained from elective terminations of viable first-trimester pregnancies (7^th^–9^th^ gestational week). Tissues were collected with written informed consent. This procedure was approved by the Ethics Committee of the Medical University of Vienna (Nr. 084/2013). For isolation of primary HDSC from first trimester decidua, tissues devoid of placental trophoblasts were selected. Sections of the respective sample were stained with antibodies against human leukocyte antigen G1 (HLA-G1) to exclude contamination with extravillous trophoblasts. Primary HDSC were prepared as previously described [21]. Briefly, decidual tissue was minced into 3 mm^3^ pieces and digested under agitation in 2 mg/ml collagenase I (Life Technologies, Paisley, UK) and 0.5 mg/ml DNase I (Sigma Aldrich, St. Louis, MO) in HBSS containing 25 mM HEPES (Life Technologies) for 30 min at 37°C. Dispersed cells were pooled, filtered through a 70 µm cell strainer, pelleted, washed in HBSS and seeded in phenolred-free DMEM/F12 medium (Invitrogen) supplemented with 10% heat-inactivated fetal bovine serum (PAA Laboratories GmbH, Pasching, Austria), 1% ITS+ Premix (BD Biosciences, San Jose, CA), and 50 µg/ml Gentamicin (Invitrogen, Carlsbad, CA) and cultivated at 37°C, 5% CO_2_ and 95% humidity.

### Differentiation of primary human decidual stromal cells

Isolated HDSC (n = 18) between passages 3 and 5 were used for in vitro decidualization as described [21]. Briefly, cells were seeded at confluence in 24-well plates (7.5×10^4^/well) and stimulated on the next day with 0.5 mM 8-Bromo-cAMP (Sigma-Aldrich) and/or 10 nM estrogen (E2; 17β-estradiol-acetate; Sigma Aldrich)/1 µM progesterone (P4; 4-pregnene-3,20-dione; SigmaAldrich) for 3 and 6 days. HDSC cultivated in the absence of cAMP or E2P4 served as non-stimulated controls. On day 3, medium was changed and stimuli were refreshed.

### Immunofluorescence

First trimester decidual tissue samples (n = 5) were fixed with 7.5% formaldehyde and embedded in paraffin (Merck, Darmstadt, Germany). Deparaffinized tissue sections (3 µm) were boiled in 1x Target Retrieval Solution, pH 6.1 (Dako, Glostrup, Denmark) and blocked with 0.05% fish skin gelatin (Sigma Aldrich), followed by incubation overnight at 4°C with the following primary antibodies: Notch1 (1∶100; Cell Signaling Technology, 3608), Notch2 (1∶100; Cell Signaling Technology, 5732), Notch3 (2 µg/ml, Santa Cruz Biotechnology, sc-5593), Notch4 (5 µg/ml; Lifespan, LS-C137135), Jagged1 (2 µg/ml; Santa Cruz Biotechnology, sc-8303), Jagged2 (1∶100; Cell Signaling Technology, 2210), DLL1 (10 µg/ml; Abcam, ab76655), DLL3 (2 µg/ml; Santa Cruz Biotechnology, sc-67269), DLL4 (17.6 µg/ml; Abcam, ab-7280), vimentin (1.44 µg/ml; GeneTex, 100619 or 1∶200, DAKO, M7020), CD45 (1∶100, DAKO, M0701), cytokeratin 7 (1.96 µg/ml; DAKO, M7018) and HLA-G1 (1∶200, Exbio, 1P-292-C100). Subsequently, sections were incubated with goat anti-mouse or anti-rabbit IgG conjugated to Alexa Fluor 488 or Alexa Fluor 568 (2 µg/ml, Molecular Probes, Life Technologies) for 1 hour at room temperature, counterstained with DAPI (1 mg/ml, Roche Diagnostics, Mannheim, Germany) and mounted in Fluoromount-G (SouthernBiotech, Birmingham, AL). Adequate IgG-control antibodies were used accordingly: mAb IgG XP Isotype Control (10 µg/ml, Cell Signaling Technology, 3900) and Normal Rabbit IgG (10 µg/ml, Cell Signaling Technology, 2729). Images were acquired on a fluorescence microscope (Olympus BX50, CC12 digital camera, Cell∧P software).

### RNA isolation and quantitative real time-PCR

Total RNA was extracted by direct lysis in the culture dishes using TriFast Reagent (PeqLab, Erlangen, D) according to the manufacturer's instructions. For RNA extraction from decidua and placenta, tissue was homogenized with Precellys24 (Bertin Technologies), the homogenate was lysed with TriFast Reagent (PeqLab, Erlangen, D). RNA amount and integrity were evaluated using the Agilent Bioanalyzer 2100 (Agilent, Palo Alto, CA). 2 µg RNA were reverse transcribed using 200U of RevertAid H Minus M.MuLV Reverse Transcriptase (Fermentas, St.Leon-Rot, D), 0.2 µg Hexanucleotide Mix (Roche Diagnostics GmbH, Vienna, Austria), 0.5 µl RNaseOUT (Recombinant Ribonuclease Inhibitor, Invitrogen) and 0.5 mM dNTP (Promega, Madison, WI) in a final volume of 20 µl. Quantitative real-time PCR was performed on the ABI 7500 Sequence Detection System (Applied Biosystems, Carlsbad, CA) using Taq Man Gene Expression Assays (TaqMan Universal PCR Master Mix, 20x Taq Man Gene Expression Assay Mix for Notch1 (Hs01062014_m1), Notch2 (Hs01050702_m1), Notch3 (Hs01128541_m1), Notch4 (Hs00965889_m1), Jagged1 (Hs01070032_m1), Jagged2 (Hs00171432_m1), DLL1 (Hs00194509_m1), DLL3 (Hs01085096_m1), DLL4 (Hs00184092_m1), HES1 (Hs00172878_m1), PRL (Hs00168730_m1), IGFBP1 (Hs00426285_m1) and TATA-box binding protein (TBP; TaqMan endogenous control)) according to the manufacturer's instructions. Signals were calculated as described previously [21].

### Western blot analysis

Isolation of total cellular protein from HDSC was performed by freezing-thawing in a cell culture lysis reagent (Promega) containing protease inhibitor cocktail (1∶100, Invitrogen). For protein isolation from decidua and placenta, tissue was homogenized with Precellys24 (Bertin Technologies), the homogenate was lysed with a cell culture lysis reagent (Promega) containing protease inhibitor cocktail (1∶100, Invitrogen). 20 µg of total protein lysates were separated on 8,5% SDS/polyacrylamide gels and blotted onto methanol-activated polyvinyliden difluoride membranes (GE Healthcare, Buckinghamshire, UK) as mentioned [22], [23]. After blocking with 5% non-fat dry milk in TBST, membranes were incubated overnight at 4°C with the following primary antibodies: Notch1 (1∶1000; Cell Signaling Technology, 4380), Notch2 (1∶1000; Cell Signaling Technology, 5732), Notch3 (1∶1000; Cell Signaling Technology, 2889), Notch4 (0.5 µg/ml; Santa Cruz Biotechnology, sc-5594), Jagged1 (0.2 µg/ml; Santa Cruz Technology, sc-8303), Jagged2 (1∶2000; Cell Signaling Technology, 2210), DLL1 (2 µg/ml; Abcam, ab76655), DLL3 (0.4 µg/ml; Santa Cruz Biotechnology, sc-67269) and DLL4 (0.88 µg/ml; Abcam, ab-7280). GAPDH (1∶2000; Cell Signaling Technology, 2118) was used as loading control. Subsequently, membranes were incubated with HRP-linked anti-rabbit IgG (1∶25,000, GE Healthcare) secondary antibody for 1 hour at room temperature. The blots were developed with ECL Prime Western blotting detection reagent (GE Healthcare) and proteins were visualized using MultiImage III FC Light Cabinet and Alpha View 3.1.1.0 software (Alpha Innotech, San Leandro, CA). Quantification of Western blots was done using Image J software.

### Inhibition and gene silencing of Notch2

For chemical inhibition of canonical Notch signaling HDSC were seeded at confluence in 24-well plates (7.5×10^4^/well) and decidualization was induced by the above mentioned stimuli in the presence of 10 µM DAPT the next day. To specifically inhibit the Notch2 receptor, seeded cells were treated with 0.4 µg/ml human IgG isotype control (Novus Biologicals) or 0.4 µg/ml therapeutic human Notch2 IgG_1_ blocking antibody, which binds and stabilizes the auto-inhibited, negative regulatory region (NRR) of the receptor, preventing conformational changes and therefore ADAM-mediated cleavage [24]. For siRNA-mediated Notch2 knockdown HDSC were seeded in 24-well plates at a density of 5×10^4^ cells per well. After 24 hours cells were transfected with each 0.5 mg/ml non-targeting control (ntc) siRNAs (D-001810-01-05) or Notch2 siRNAs (L-012235-00; ON-TARGETplus SMARTpools, Dharmacon-Thermo Fisher Scientific, MA) mixed with Lipofectamine RNAiMAX transfection reagent (Invitrogen) according to the manufacturer's instructions. After 24 hours of incubation with either blocking antibody or siRNA, cAMP and E2P4 were added to induce decidualization. Treatments were refreshed on day 4. Supernatants, cellular protein and RNA were harvested at indicated time points.

### Luciferase assay

For detection of Notch activity, sub-confluent HDSC were co-transfected with 1 µg/ml of a luciferase reporter plasmid containing four RBPJκ binding sites [25] and 0.5 µg/ml pCMV-β-galactosidase (CMV-βGal; normalization control) for 6 hours using 2 µg/ml Lipofectamine 2000 according to the manufacturer's instructions (Invitrogen). Cells were cultured overnight in the absence or presence of 10 µM N-[N-(3,5-Difluorophenacetyl-L-alanyl)]-S-phenylglycine t-Butyl Ester (DAPT; Calbiochem). Alternatively, transfected HDSC were incubated with the specific Notch2 blocking antibody and IgG control for 24 hours as described above. Subsequently, cultures were incubated with cAMP and E2P4 for another 24 hours. Proteins were harvested using the above mentioned cell culture lysis buffer (Promega). Luciferase and β-galactosidase activities were determined as previously indicated [22], [23].

### ELISA

PRL levels in culture supernatants of HDSC were determined using a sandwich-type ELISA (25-PROHU-E01, ALPCO Diagnostics, Salem, NH) according to the manufacturer's instructions. PRL protein concentrations were normalized to cellular protein content measured by using the Bio-Rad Protein assay (Bio-Rad Laboratories, CA).

### Statistical analyses

For multiple comparison, statistical analyses were performed with Anova and appropriate post hoc tests (Games-Howell or Dunett's T3) using SPSS 17 (SPSS Inc., Chicago, IL). For comparison of two conditions of interest, statistical analyses were performed with Student's paired t-test. Gaussian distribution and equality of variances were examined with Kolmogorov-Smirnov test and Levene's test, respectively. A p-value of ≤0.05 was considered statistically significant.

## Results

### Distribution of Notch receptors and ligands in first trimester decidual tissue

**Figure 1:**
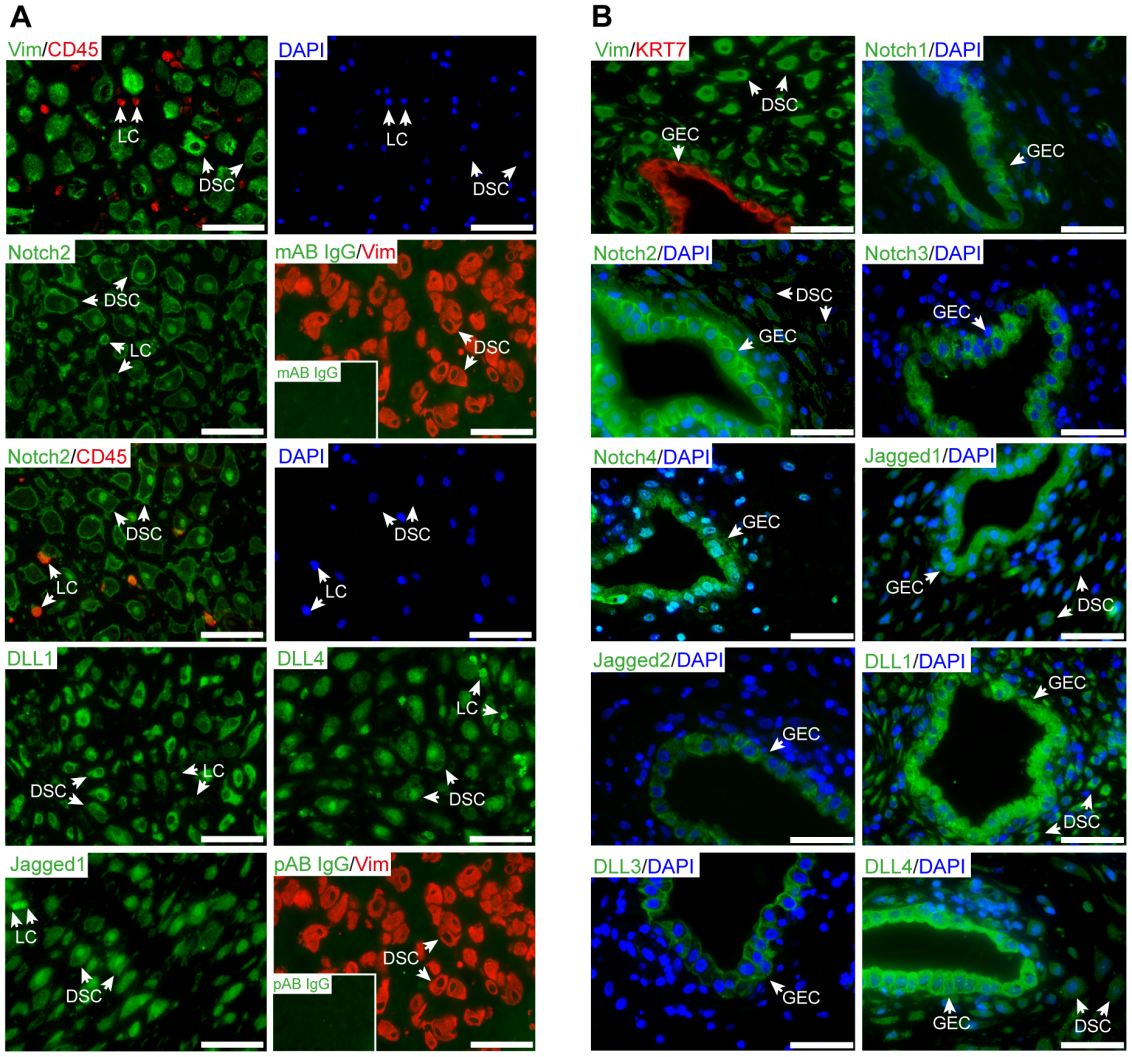
Expression pattern of Notch ligands and receptors in first trimester decidual tissue. Serial sectioning of paraffin-embedded tissues and immunofluorescence were performed as described in materials and methods. Representative examples (8^th^ week of pregnancy) of 5 different deciduae analyzed are depicted. DSC, decidual stromal cells; GEC, glandular epithelial cell; LC, leukocytes; Scale bars represent 100 µm. (A) Localization of Notch family members in human deidual stromal cells. Double staining with antibodies recognizing vimentin (Vim, green), Notch2 (green) and CD45 (red) mark decidual stromal cells and leukocytes, respectively. The respective counterstaining with DAPI is depicted on the right hand side. Double stainings with the appropriate isotype-specific monoclonal (mAb, green, insert picture) and polyclonal (pAb, green, insert picture) controls with vimentin (Vim, red) are shown. (B) Glandular expression of Notch receptors and ligands. Double staining with antibodies recognizing vimentin (Vim, green) or cytokeratin 7 (KRT7, red) was used to depict DSC and GEC, respectively. Co-staining of Notch receptor or ligand (both green) with nuclear staining (DAPI, blue) is shown.

Immunofluorescence of first trimester decidua revealed expression of Notch2, Jagged1, DLL1 and DLL4 in human decidual stromal cells (Fig. 1A). The latter could easily be distinguished from decidual leukocytes as shown by double-staining with antibodies recognizing vimentin and CD45, respectively. By utilization of a Notch2 antibody recognizing both the membrane bound and translocated NICD, Notch2 was detectable at the cell surface as well as in nuclei of stromal cells, suggesting active, canonical Notch signaling. The Notch ligands Jagged1, DLL1, and DLL4 displayed cytoplasmic staining in stromal cells whereas Jagged1 and DLL4 additionally localized to nuclei. Notch1, 3 and 4 as well as the ligands Jagged2 and DLL3 were absent from these cells, but could be detected in the glandular epithelium expressing all Notch receptors and ligands (Fig. 1B). Compared to stromal cells, glandular epithelial cells showed stronger immunofluorescence signals, particularly for Notch2 and Jagged1. Besides Notch2, Jagged1, DLL1 and DLL4 (Fig. 1A), CD45-positive leukocytes additionally expressed Notch1, 3, and 4 but lacked Jagged2 and DLL3 (Fig. S1).

### Expression of Notch receptors and ligands in differentiating HDSC

**Figure 2:**
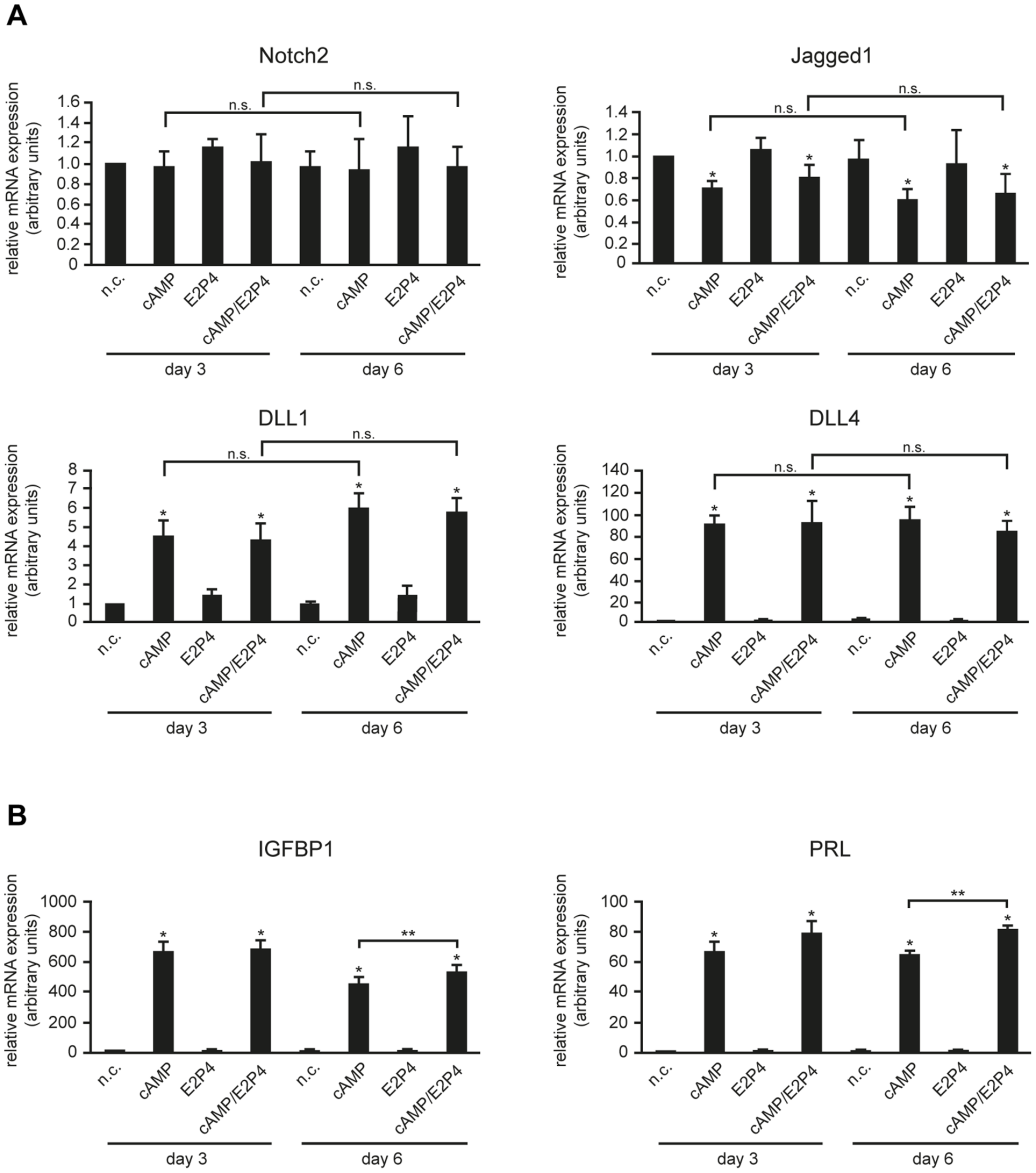
qPCR measuring mRNA expression of Notch2 receptor and Notch ligands (A) and decidual markers IGFBP1 and PRL (B) in differentiating HDSC. Cultures were incubated for 3 and 6 days with cAMP, E2P4 or cAMP/E2P4. Cells without stimuli were cultivated in parallel representing non-stimulated controls (n.c.). For relative quantification of mRNA expression, n.c. of day 3 was arbitrarily set to 1. Bars depict mean values ± S.D. of 4 different experiments. PCR reactions were performed in duplicates. * depicts p≤0.05 compared to the n.c. of day 3; n.s., not significant. ** indicates p≤0.05 between cAMP and cAMP/E2P4-treated cultures at day 6.

**Figure 3:**
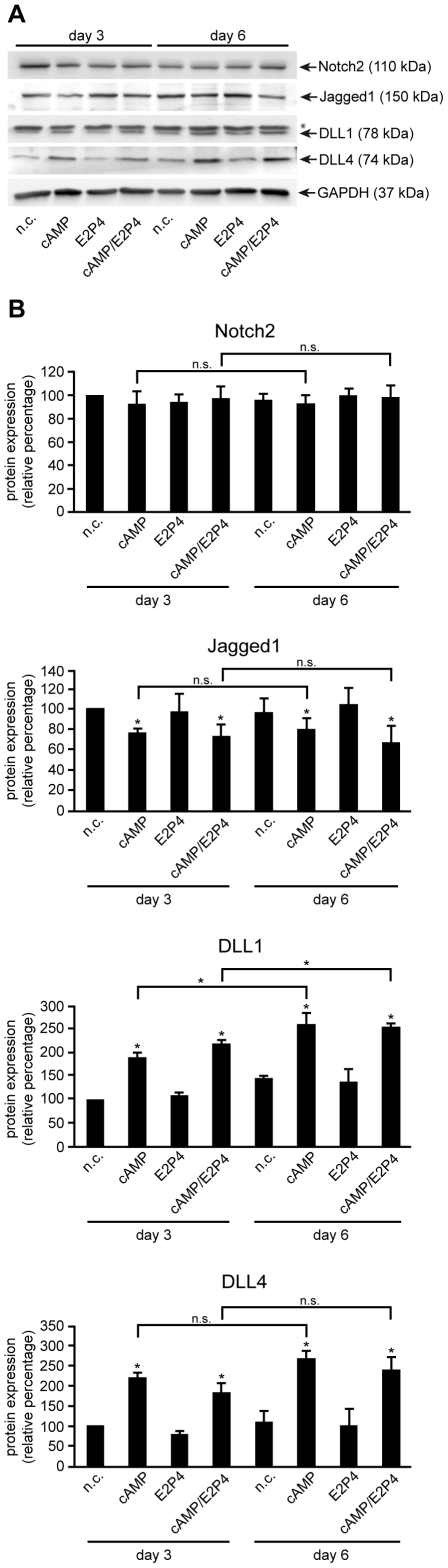
Differentiation-dependent protein expression of Notch2 receptor and Notch ligands in HDSC. Cells were stimulated with cAMP and/or E2P4 for 3 and 6 days. Total protein lysates were analyzed by western blotting and quantitated using densitometry as described in Materials and methods. A representative example out of 3 different experiments is shown. n.c., non-stimulated controls; (A) Western blot showing specific signals for Notch2, Jagged1, DLL1 and DLL4 (marked by arrows). GAPDH was used as a loading control, * indicates unspecific band. (B) Densitometrical quantification of western blot signals normalized to GAPDH. Bars (n = 3) represent mean values ± S.D. For relative quantification of protein expression, values of n.c. at day 3 were arbitrarily set to 100%. * indicates p≤0.05 compared to n.c. of day 3; n.s., not significant.

To verify the decidual stromal cell-specific expression observed in tissue sections, Notch receptors and ligands were studied in isolated, primary HDSC which, similar to endometrial stromal cells, differentiate in vitro in the presence of estrogen/progesterone (E2P4) and/or cAMP [21]. To monitor the dynamic expression of Notch receptors and ligands, transcript and protein levels of Notch2, Jagged1, DLL1 and DLL4 were analyzed at day 3 and 6 of decidualization using qPCR (Fig. 2A) and Western blotting (Fig. 3A), respectively. Additionally, mRNA expression of the decidual markers IGFBP1 and PRL was assessed to ensure proper in vitro decidualization. As expected, IGFBP1 and PRL transcript levels were strongly induced upon treatment with cAMP, however treatment of cAMP in combination with E2P4 proved to be the most effective stimulus for in vitro decidualization (Fig. 2B). Quantitative real time analyses showed constitutively expressed Notch2 mRNA levels after 3 and 6 days of treatment with E2P4 and/or cAMP (Fig. 2A). Compared to untreated cultures, Jagged1 was weakly downregulated in the presence of cAMP alone or in combination with E2P4, but not upon sole hormonal treatment. In contrast, addition of cAMP and the combined treatment increased DLL1 mRNA levels 5.1 and 5.3 fold at day 3 of cultivation, whereas DLL4 was elevated 94 and 95 fold, respectively. The presence of E2P4 alone did not affect mRNA expression of these ligands. Protein levels of Notch family members mirrored mRNA expression patterns in differentiating HDSC (Fig. 3). While Notch2 did not show stimuli- or time-dependent regulation, Jagged1 was faintly diminished upon incubation with cAMP or cAMP/E2P4 (Fig. 3B). In contrast, cAMP or the combined treatment increased protein expression of DLL1 and DLL4 in HDSC. Moreover, cAMP- or cAMP/E2P4-induced DLL1 protein levels differed significantly between 3 and 6 days of cultivation, respectively. However, Notch1, 3 and 4 as well as Jagged2 and DLL3 mRNA and protein were undetectable in HDSC using real-time PCR and Western blotting (unpublished observation), confirming results obtained in the immunofluorescence analyses (Fig. 1B and Fig. S2).

### HDSC exhibit increased canonical Notch activity during in vitro differentiation

Compared to single treatments with cAMP or E2P4, HDSC were shown to express elevated levels of the decidual marker genes PRL and IGFBP1 upon addition of both cAMP and E2P4, suggesting that the combined treatment represents the most physiological stimulus for functional differentiation [21]. Since E2P4 treatment had no effects on the expression of Notch signaling components (Fig. 2 and Fig. 3), subsequent experiments were only performed in the presence of both cAMP and E2P4.

**Figure 4:**
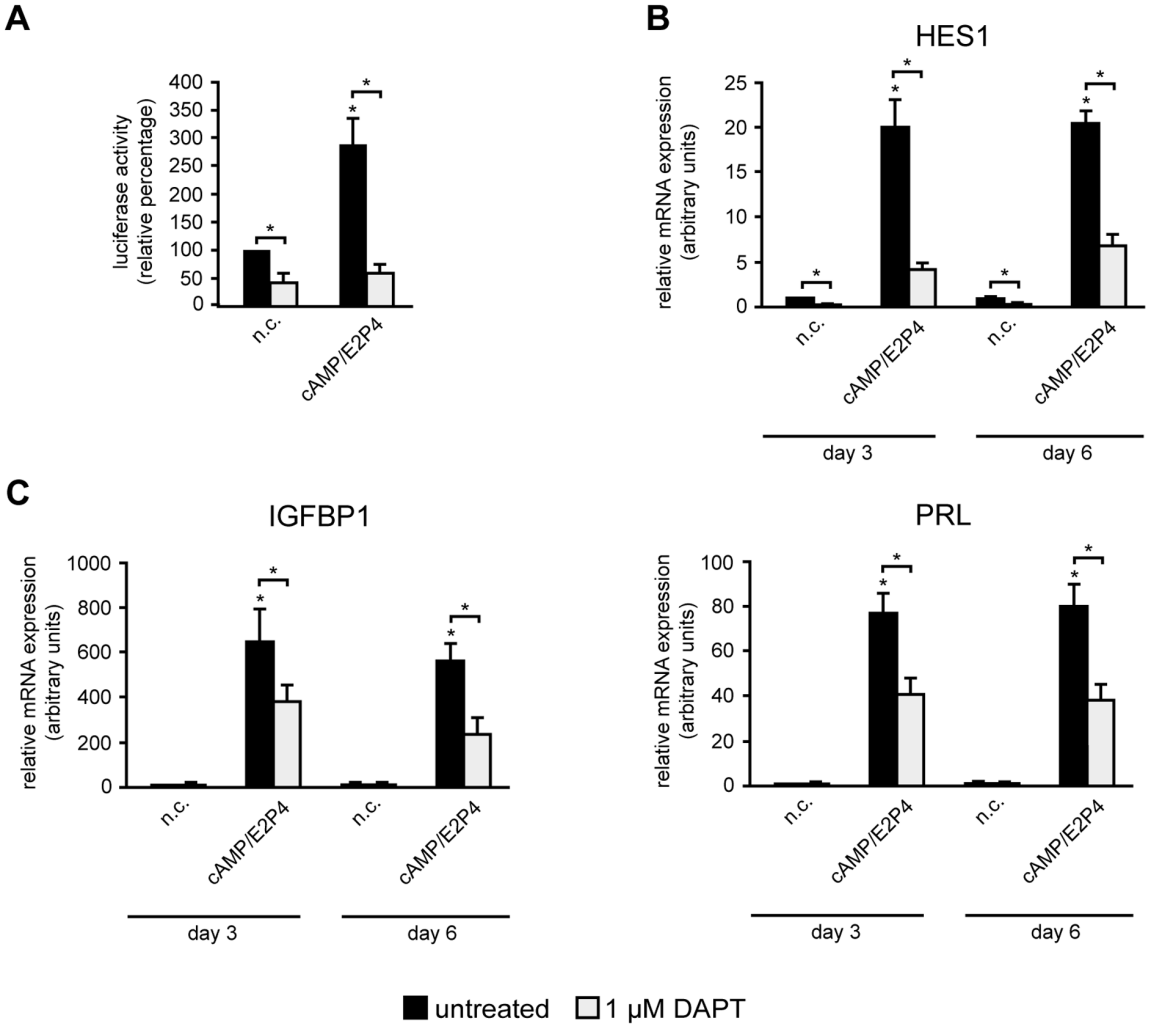
Inhibition of canonical Notch signalling impairs decidualization of HDSC. Stimulation with cAMP/E2P4 in the absence or presence of the γ-secretase inhibitor DAPT, transfection of the RBPJκ luciferase-reporter plasmid and quantitative real time PCR were performed as described in materials and methods. n.c. non-stimulated controls. (A) Luciferase activity of a canonical Notch reporter plasmid harboring RBPJκ cognate sequences normalized to constitutive β-Gal expression after treatment with decidualizing stimulus and DAPT. Mean values ± S.D. of 3 experiments performed in duplicates are shown. Values of n.c. transfected with RBPJκ plasmids in the absence of DAPT were arbitrarily set at 100%. (B, C) Quantitative real-time PCR analyses evaluating mRNA expression of the Notch target gene HES1 (B) or marker genes of differentiation, IGFBP1 and PRL (C), in decidualizing cultures in the absence or presence of DAPT. For relative quantification of mRNA expression n.c. at day 3 (absence of DAPT) was arbitrarily set to 1. Bars indicate mean values ± S.D. of 3 different experiments. PCR reactions were performed in duplicates. * indicates p≤0.05 compared to n.c. of day 3. Significant changes (*, p≤0.05) between DAPT-treated (open bars) and untreated (black bars) cells are indicated by brackets.

To assess Notch activity during HDSC differentiation, cultures were transfected with a canonical Notch luciferase reporter plasmid, containing 4 binding sites for the crucial NICD-dependent transcription factor RBPJκ, and analyzed in the absence or presence of cAMP/E2P4. The decidualizing stimulus increased basal luciferase activity 2.5 fold, whereas reporter expression was significantly suppressed in the presence of the Notch/γ-secretase inhibitor DAPT, both in non-stimulated and differentiating cultures (Fig. 4A). Quantitative qPCR analyses of the Notch target gene HES1 (Fig. 4B) revealed that mRNA levels were enhanced in cultures treated with cAMP/E2P4, but downregulated upon chemical inhibition with DAPT. To further investigate the impact of Notch signaling on decidualization, two critical marker genes of differentiation, IGFBP1 and PRL, were measured by qPCR (Fig. 4C). Supplementation of cAMP/E2P4 strongly increased IGFBP1 and PRL transcript levels at day 3 and 6 of HDSC differentiation. However, treatment with DAPT significantly decreased cAMP/E2P4-induced IGFBP1 and PRL mRNA levels, suggesting that canonical Notch signaling contributed to differentiation-dependent marker gene expression.

### siRNA-mediated gene silencing and antibody-mediated inhibition of Notch2 in HDSC

**Figure 5:**
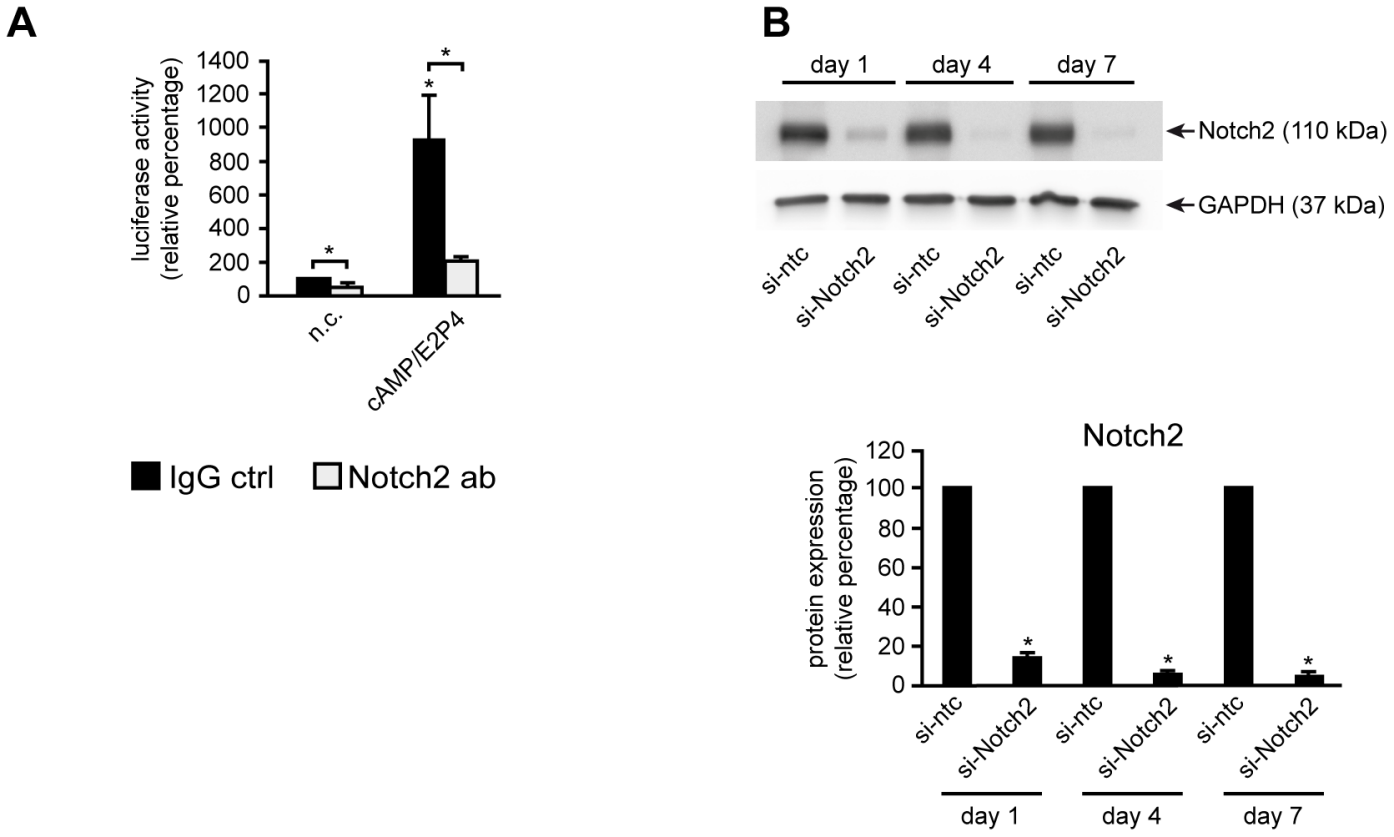
Downregulation of canonical Notch activity and Notch2 protein expression in HDSC. Cells were cultured in the absence or presence of Notch2 blocking antibodies (Notch2 ab) (A) or si-RNAs targeting Notch2 (si-Notch2) (B). (A) Luciferase activity of the canonical Notch reporter in the presence of Notch2 ab or IgG controls. Reporter expression was normalized to constitutive β-Gal activity. Mean values ± S.D. of 3 experiments performed in duplicates are shown. IgG ctrl, IgG control; * indicates p≤0.05 compared to IgG-treated n.c. Significant changes (*, p≤0.05) between DAPT-treated (open bars) and untreated (black bars) cells are indicated by brackets. (B) Western blot showing Notch2 protein expression in siRNA-treated HDSC. Specific Notch2 signals are marked by an arrow (110 kDa). GAPDH was used as a loading control. Representative examples of 3 independent experiments are depicted. Lower panel shows the densitometrical quantification calculated from 3 independent experiments. si-ntc, si-non targeting control. * indicates p≤0.05 compared to si-ntc of the same day.

Data shown above suggested that canonical Notch signaling could regulate decidual differentiation via Notch2, since the latter was the only receptor present in decidual stromal cells of tissues and in isolated HDSC. To verify this hypothesis, specific Notch2 blocking [24] or IgG control antibodies were added to HDSC transfected with the canonical Notch reporter (Fig. 5A). Analyses of luciferase activity revealed that, similar to the DAPT treatment, basal and cAMP/E2P4-induced Notch reporter expression was suppressed in the presence of Notch2 blocking antibodies. As a second approach to abolish Notch activity in HDSC, Notch2 was downregulated by siRNA-mediated gene silencing (Fig. 5B). Western blotting and densitometrical quantification of signals indicated maximal downregulation of Notch2 after 7 days of incubation with the specific siRNA.

### Inactivation of Notch2 signaling impairs differentiation of HDSC

**Figure 6:**
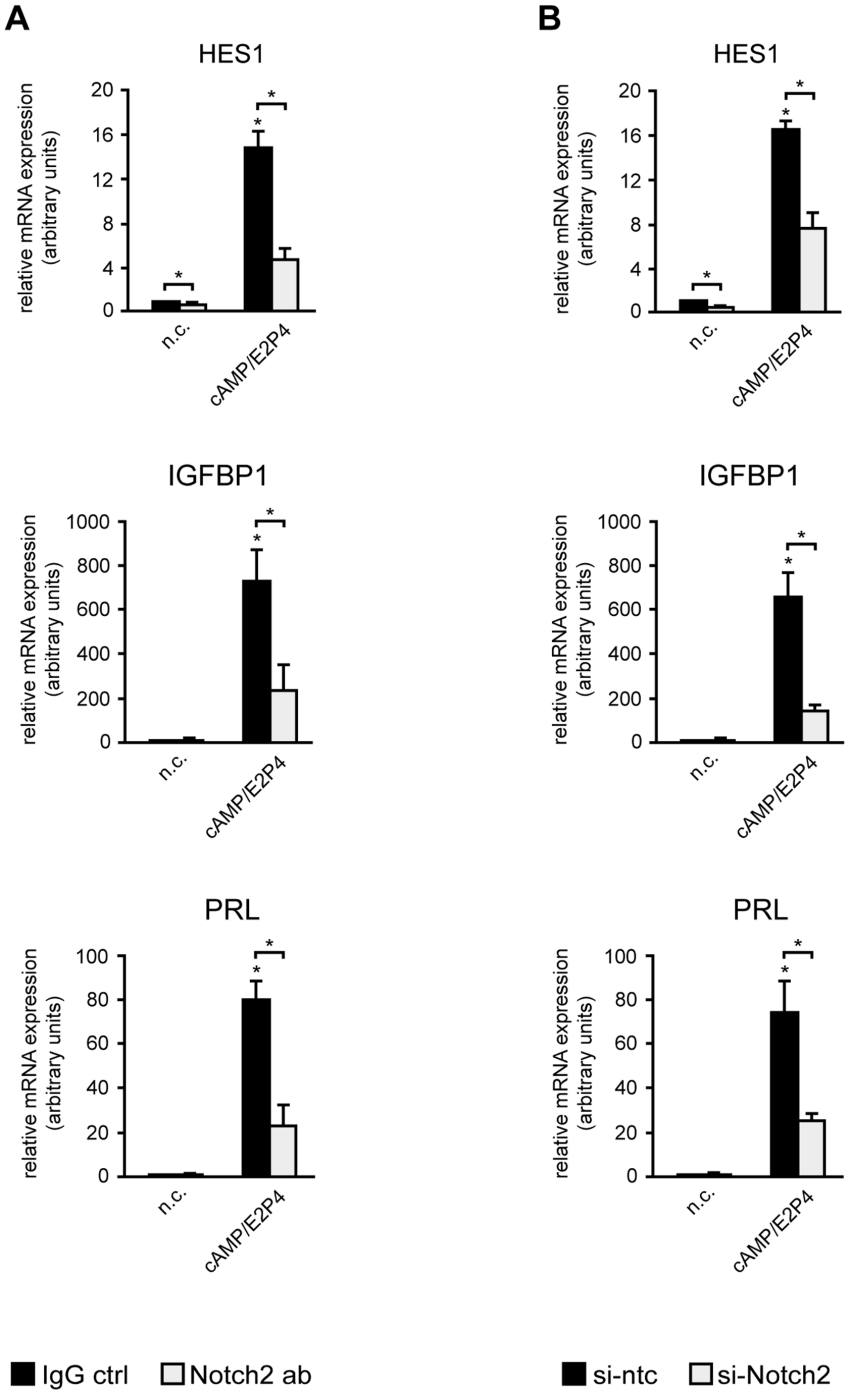
mRNA expression in HDSC after antibody-mediated inhibition or siRNA-mediated downregulation of Notch2. HDSC were differentiated with cAMP/E2P4 for 6 days in the absence or presence of Notch2-blocking antibodies or siRNAs as mentioned in materials and methods. Quantitative real-time PCR analyses showing mRNA expression of the Notch target gene HES1 and the markers of decidualization, IGFBP1 and PRL, upon addition of Notch2 blocking antibodies (A) or Notch2 siRNAs (B). For relative quantification non-stimulated controls (n.c.) were arbitrarily set to 1. Bars indicate mean values ± S.D. of 3 different experiments performed in duplicates. * indicates p≤0.05 compared to n.c.; Significant changes (*, p≤0.05) between Notch2 ab or si-Notch2-treated cells (open bars) and untreated (black bars) cells are indicated by brackets. IgG ctrl, IgG control; si-ntc, si-non targeting control.

**Figure 7:**
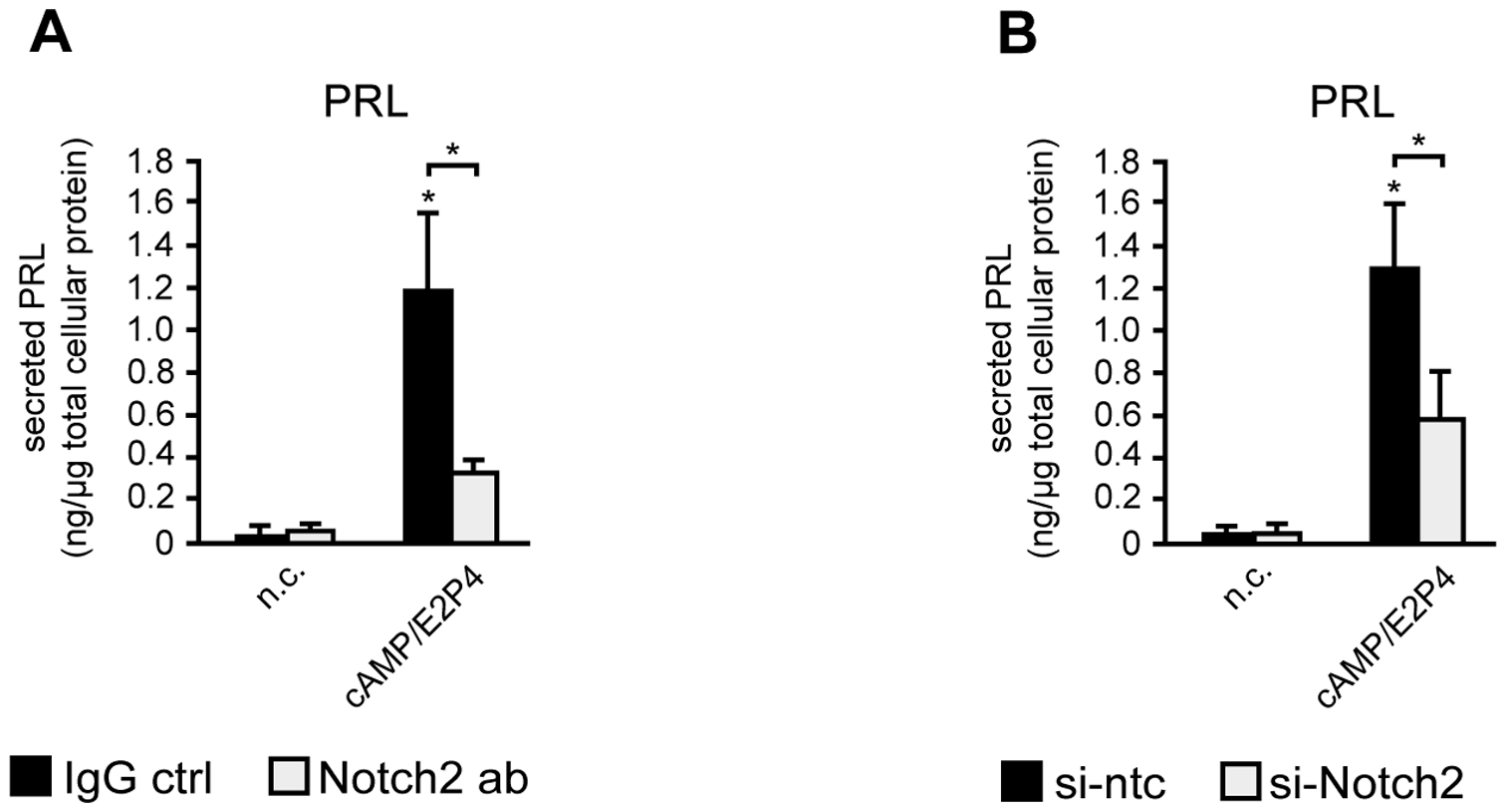
Notch2 inhibition diminishes differentiation-dependent prolactin secretion in HDSC. Cells were incubated with Notch2 blocking antibodies (A) or Notch2 siRNAs (B) and differentiated for 6 days in the presence of cAMP/E2P4. Supernatants were collected and prolactin (PRL) concentrations were measured by ELISA. Normalization to cellular protein concentrations was performed as mentioned in materials and methods. Bars represent mean values ± S.D. of each 3 different experiments performed in duplicates. * indicates p≤0.05 compared to non-stimulated control (n.c.); Significant changes (*, p≤0.05) between Notch2 ab or si-Notch2-treated cells (open bars) and untreated (black bars) cells are indicated by brackets. IgG ctrl, IgG control; si-ntc, si-non targeting control.

To evaluate the role of Notch2 in HDSC differentiation, the Notch target gene HES1, as well as the decidualization markers IGFBP1 and PRL were analyzed in Notch2 blocking antibody- or siRNA-treated cultures (Fig. 6). Cultures were analyzed at day 6, when optimal gene silencing of Notch2 was achieved. Analyses using qPCR revealed a downregulation of HES1 transcript levels upon addition of Notch2 blocking antibodies (Fig. 6A) or siRNAs (Fig. 6B) both in undifferentiated and decidualizing cultures. Similarly, supplementation of Notch2 blocking antibodies (Fig. 6A) or incubation with Notch2 siRNAs (Fig. 6B) significantly decreased cAMP/E2P4-induced IGFBP1 and PRL mRNA levels. Along those lines, addition of Notch2 blocking antibodies (Fig. 7A) or siRNAs (Fig. 7B) significantly reduced PRL protein levels in supernatants of cAMP/E2P4-treated HDSC, again suggesting impaired differentiation and decidual marker gene expression when Notch2-dependent signaling was inhibited.

## Discussion

Canonical Notch signaling is critically involved in development and tissue homeostasis controlling diverse cellular processes such as stem cell maintenance, proliferation, differentiation or apoptosis [8]. Therefore, it may not be surprising that the particular pathway also regulates reproductive processes in different species. Notch receptors and ligands are differentially expressed and dynamically regulated in distinct compartments of the developing mouse placenta [26]. Homozygous mutations in mice revealed that Notch signaling is involved in placental branching morphogenesis, placental vasculogenesis, endovascular trophoblast invasion as well as uterine stromal cell differentiation [27], [28], [29], [30]. Moreover, using baboon as a model, pregnancy hormone-dependent induction of Notch1 was shown to be critical for survival and differentiation of endometrial stromal cells [17].

However, the role of the particular signaling pathway in human reproductive processes is largely unknown. Expression of Notch signaling components has been investigated in different cell types of the cycling endometrium as well as in term placental tissues with considerable divergence between studies [28]. Along those lines, expression patterns of Notch receptors and ligands in human trophoblast subtypes of early pregnancy have only recently been unraveled, whereas distributions in different first trimester decidual cells have not been analyzed [22], [28]. Hence, the present study was conducted to gain more insights into the expression patterns of Notch signaling components in the decidua of early pregnancy and their putative roles in decidualization. Previous publications showed expression of all Notch receptors, DLL4 and Jagged1 in glandular epithelial cells of the endometrium, but other ligands have not been investigated [12], [13], [15]. Immunofluorescence analyses of early pregnancy tissue indicated that glandular epithelial cells of the decidua expressed the four Notch receptors, DLL4 and Jagged1, and in addition DLL1, 3, and Jagged2. Therefore, we speculate that the Notch pathway could be important for glandular development and/or function. Decidual NK cells were shown to produce Notch1 and Notch2 but lacked Notch3 and 4 [18]. So far, expression of Notch receptors and ligands in other immune cells of the decidua has not been investigated. Data of this study indicated that CD45-positive cells express all Notch receptors as well as DLL1, 4 and Jagged1 suggesting that the Notch pathway could be required for coordinated interactions of leukocytes with other cell types of the decidua. However, it was not intended to further characterize immune cell subtype-specific expression patterns of Notch and its ligands herein.

Immunofluorescence, quantitative PCR and Western blot analyses revealed that HDSC of first trimester pregnancy expressed Notch2, Jagged1, DLL1, and DLL4 mRNA and protein both in vivo and during in vitro cultivation, whereas other receptors and ligands were absent. Previous immunofluorescence analyses failed to detect Jagged1 and DLL4 in maternal uterine cells of second trimester pregnancies [29] suggesting downregulation of these ligands during gestation. Furthermore, the present data indicate that Notch2, the only receptor produced in first trimester HDSC, plays a role in the decidual differentiation process. Besides its localization at the cell membrane, Notch2 was also detectable in nuclei of these cells suggesting nuclear recruitment of Notch2ICD and therefore activation of canonical Notch signaling. Interestingly, the ligands Jagged1 and DLL4 also partly localized to nuclei of HDSC. Similar to the Notch receptors, DSL ligands can be cleaved by ADAM proteins and γ-secretase resulting in ligand shedding and/or generation of intracellular domains (ICDs) [31]. The latter translocate into the nucleus via specific nuclear localization signals (NLSs) or positively charged residues adjacent to the transmembrane domain [31]. Little is known about specific functions of Notch ligand ICDs. However, it was shown that they can act as co-activators of SMAD-dependent transcription [32]. Since TGFβ-SMAD signaling plays a role in decidualization [33], [34], it may well be that ICDs of Jagged1 and/or DLL4 are also involved in the differentiation process. In this context, it is also noteworthy that mRNA and protein of DLL1 and DLL4 are induced during in vitro cultivation of HDSC with cAMP/E2P4. Hormones such as relaxin, promoting decidualization through cAMP, were also shown to stimulate DLL4 expression in endometrial epithelial cells [14], [35]. Therefore, upregulation of these ligands and subsequent enforcement of Notch2-DLL1 and/or Notch2-DLL4 interactions could be a major cause for activation of canonical Notch signaling in HDSC during in vitro decidualization. Indeed, treatment of HDSC with cAMP/E2P4 increased transcriptional activity of a canonical Notch reporter as well as mRNA expression of the Notch target gene HES1.

In a recent study we could show that, similar to its effects on human endometrial stromal cells [2], cAMP provoked morphological changes of isolated HDSC as well as induction of the decidual markers PRL and IGFBP1, whereas E2P4 did not alter cellular appearance and increased PRL and IGFBP1 only upon long-term treatment [21]. Since addition of cAMP for 9 to 12 days considerably decreased HDSC numbers, likely due to its apoptotic effects, 6 days of cultivation were chosen as the optimal time point for a combined treatment with cAMP and E2P4. Whereas cAMP/E2P4 elevated the decidual marker genes PRL and IGFBP1 compared to cAMP alone [21], the combined treatment did not further increase DLL1, DLL4 or HES1 expression in the present study. Hence, the data suggest that cAMP is the main trigger for the activation of canonical Notch activity during HDSC differentiation. Besides upregulation of Notch ligands, other mechanisms might contribute to cAMP-dependent induction of the particular signaling pathway. Activation of γ-secretase, expression of transcriptional co-activators, or elevated production of RBPJκ could play a role [36], [37], [38], although the latter could not be observed in HDSC (unpublished observation). Reduction of basal HES1 expression as well as canonical Notch reporter activity upon silencing or blocking of Notch2 in non-stimulated cells suggested that the canonical pathway is active in HDSC. However, additional effects of ligand-independent, cAMP-induced Notch signaling cannot be ruled out.

Recent data suggested that an immediate P4 response occurred in HDSC, as induction of Dickkopf-1 (DKK1), a soluble inhibitor of canonical Wingless (Wnt) signaling, could be observed after 3 days of incubation [21]. DKK1 is also transcriptionally controlled by P4 in endometrial stromal cells [39]. Herein, neither 3 nor 6 days of cultivation with E2P4 stimulated mRNA and protein expression of Notch signaling components, although hormone-induced DKK1 mRNA levels could be detected in these cells [39], providing further evidence that cAMP is the main regulator of canonical Notch activity during HDSC differentiation.

Treatment of our primary cultures with the chemical Notch inhibitor DAPT indicated that HDSC exhibited basal as well as cAMP/E2P4-inducible Notch activity. Interestingly, addition of the inhibitor did not only decrease Notch reporter activity and HES1 expression, but also differentiation-induced IGFBP1 and PRL transcript levels, suggesting that the canonical Notch pathway to some extent controls expression of these prime markers of decidualization. However, besides blocking the cleavage of Notch receptors, chemical inhibition of γ-secretase may also produce side effects such as changes in the generation of other ICDs derived from N-cadherin, ERBB4 or CD44, or the release of secreted proteins from the membrane [40]. Therefore, antibody-mediated blocking and siRNA-mediated gene silencing of Notch2 was performed to specifically target the canonical Notch pathway in HDSC. Indeed, similar to the effects of DAPT treatment, both inhibitory strategies decreased mRNA expression of HES1, IGFBP1 and PRL as well as PRL protein levels in supernatants of HDSC suggesting that the canonical Notch pathway, mediated through Notch2, is involved in decidual gene expression. How Notch2 exactly controls IGFBP1 and PRL expression awaits further investigations. However, the presence of Notch2 in nuclei of decidual stromal cells in situ and the fact that antibody-mediated blocking or gene silencing of Notch2 decreased transcript levels of the decidual marker genes suggested that Notch2ICD is involved. The particular ICD could activate RBPJκ or alternatively interact with other transcription factors potentially controlling decidual gene expression such as TGFβ-dependent SMADs, NFκB or hypoxia inducible factor 1α [10], [33], [34], [41].

In conclusion, the present data show that Notch2 controls expression of markers genes of decidual differentiation in HDSC of early pregnancy. Treatment with cAMP/E2P4 increased IGFBP1 and PRL expression, the Notch ligands DLL1 and DLL4 as well as canonical Notch activity. Besides stimulation of the Notch pathway in HDSC, we speculate that elevated expression of DLL1 and DLL4 could also play a role in controlling decidual NK cell and/or trophoblast function. Indeed, decidual NK cells can interact with both DLL1 and DLL4, thereby regulating interferon-γ secretion, and DLL4 was recently shown to restrain migration of extravillous trophoblasts [18], [22]. Further studies are needed to characterize the role of Notch signaling in the complex interplay between invading trophoblasts, uNK cells, and decidual stromal cells of early pregnancy.

## Supporting Information

Figure S1 **Leukocyte-specific expression of Notch ligands and receptors in first trimester decidua (8^th^ week).** Serial sectioning of paraffin-embedded tissues and immunofluorescence protein were performed as described in Materials and methods. Representative examples of 5 different deciduae analyzed are shown. DSC, decidual stromal cells; LC, leukocyte; Double staining (upper left picture) with antibodies recognizing vimentin (Vim, green) or CD45 (red) was used to mark DSC and LC, respectively. Left panel depicts Notch receptor expression (green), whereas right panel shows the respective CD45 co-staining (red) together with DAPI (blue). Notch1, 3 and 4 are expressed in a subset of CD45-positive leukocytes. Scale bars represent 50 µm.
(TIF)

Figure S2 **qPCR measuring mRNA expression of receptors Notch1, 3, 4, and ligands Jagged2 and DLL3 in differentiating HDSC, total first trimester decidual and placental tissue.** HDSC cultures were incubated for 3 and 6 days with cAMP, E2P4 or cAMP/E2P4. Cells without stimuli were cultivated in parallel representing non-stimulated controls (n.c.). For relative quantification of mRNA expression, signals obtained for total decidua were arbitrarily set to 1. Bars depict mean values ± S.D. of 4 different experiments. PCR reactions were performed in duplicates.
(TIF)
