# Supplementary figures and images for: Notch2 Controls Prolactin and Insulin-Like Growth Factor Binding Protein-1 Expression in Decidualizing Human Stromal Cells of Early Pregnancy
Source: PLoS One. 2014 Nov 14;9(11):e112723. doi: 10.1371/journal.pone.0112723 (PMC4232464; doi:10.1371/journal.pone.0112723)

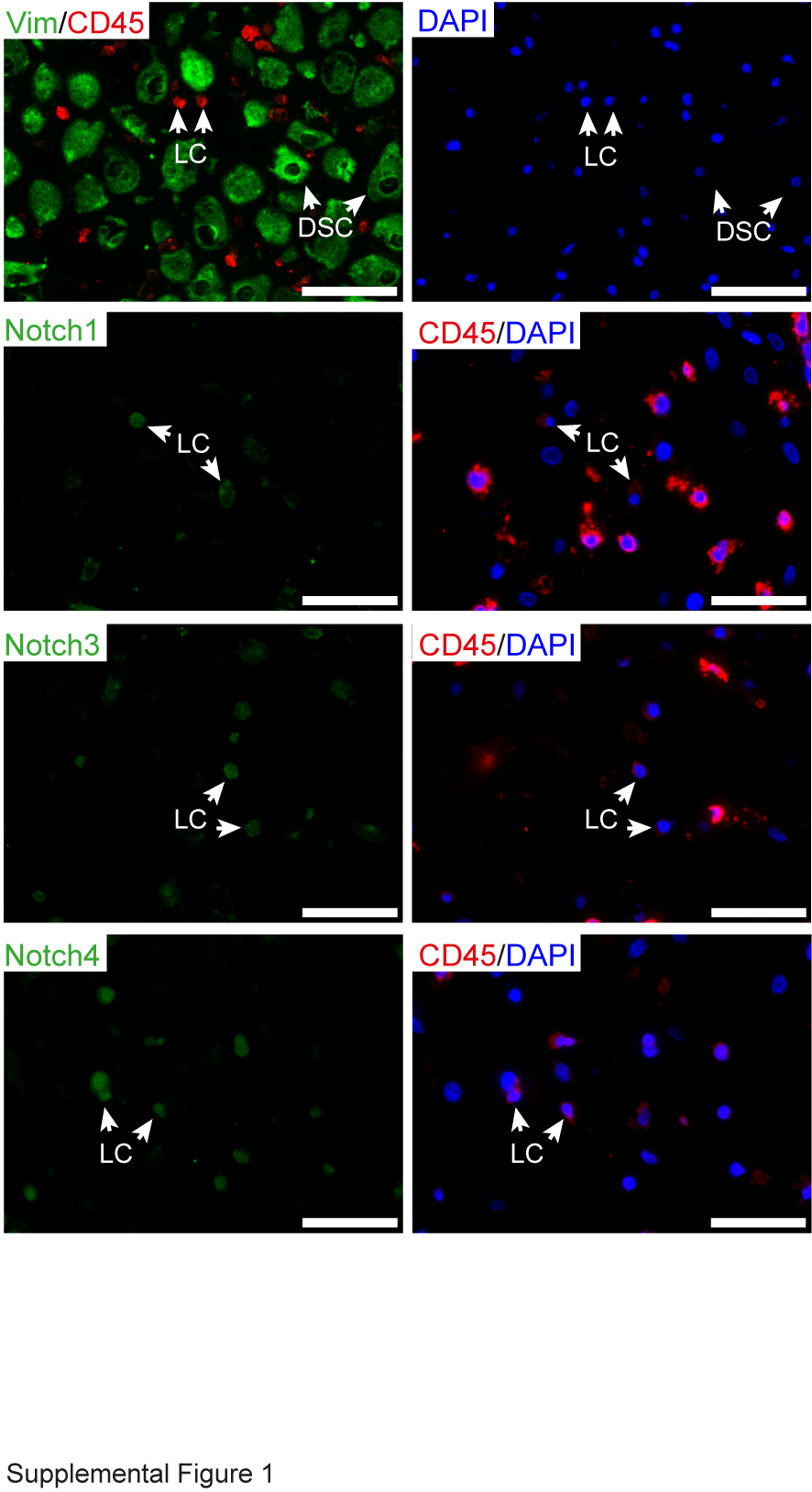

Supplement: Figure S1 — Leukocyte-specific expression of Notch ligands and receptors in first trimester decidua (8th week). Serial sectioning of paraffin-embedded tissues and immunofluorescence protein were performed as described in Materials and methods. Representative examples of 5 different deciduae analyzed are shown. DSC, decidual stromal cells; LC, leukocyte; Double staining (upper left picture) with antibodies recognizing vimentin (Vim, green) or CD45 (red) was used to mark DSC and LC, respectively. Left panel depicts Notch receptor expression (green), whereas right panel shows the respective CD45 co-staining (red) together with DAPI (blue). Notch1, 3 and 4 are expressed in a subset of CD45-positive leukocytes. Scale bars represent 50 µm. (TIF) [file pone.0112723.s001.tif]

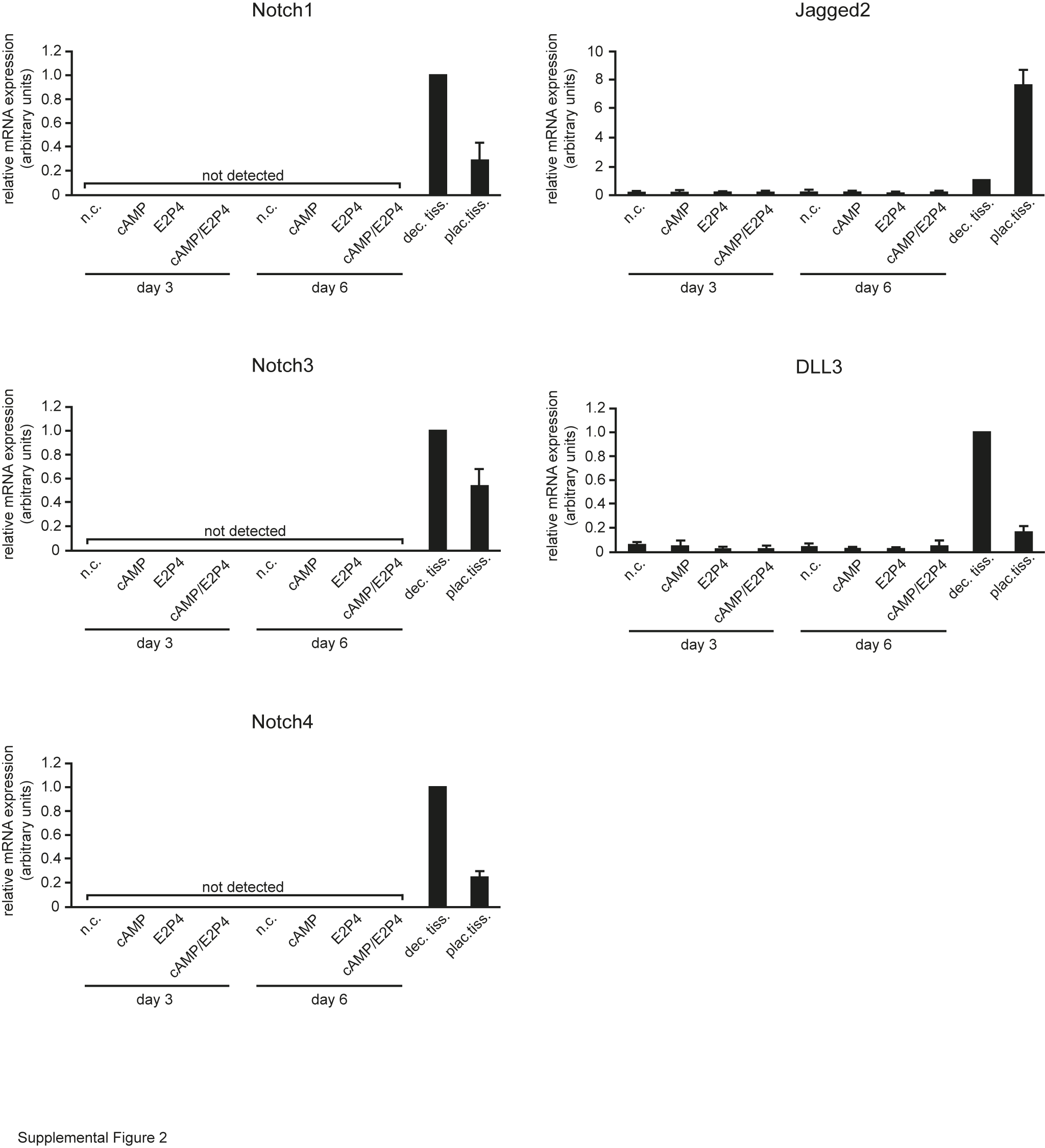

Supplement: Figure S2 — qPCR measuring mRNA expression of receptors Notch1, 3, 4, and ligands Jagged2 and DLL3 in differentiating HDSC, total first trimester decidual and placental tissue. HDSC cultures were incubated for 3 and 6 days with cAMP, E2P4 or cAMP/E2P4. Cells without stimuli were cultivated in parallel representing non-stimulated controls (n.c.). For relative quantification of mRNA expression, signals obtained for total decidua were arbitrarily set to 1. Bars depict mean values ± S.D. of 4 different experiments. PCR reactions were performed in duplicates. (TIF) [file pone.0112723.s002.tif]
